# Reply

**DOI:** 10.1016/j.jvsv.2025.102305

**Published:** 2026-01-08

**Authors:** Eri Fukaya

**Affiliations:** Division of Vascular Surgery, Stanford School of Medicine, Palo Alto, CA

Diabetic foot ulcers (DFUs) remain one of the most intractable chronic wounds and a complication of diabetes, putting a heavy burden on patients and health systems alike. The combination of chronic inflammation, infection, and impaired tissue repair can produce wounds that persist for months, sometimes years. The review by Feng and Ju^1^ takes aim at the immunologic underpinnings of this problem, spotlighting four inflammatory pathways—prostaglandin I_2_ receptor (PGI_2_), C-X-C motif chemokine receptor 4 (CXCR4), colony-stimulating factor 2 receptor α (CSF2RA), and colony-stimulating factor 3 receptor (CSF3R)—as potential therapeutic targets.^1^ This critical shift involves moving away from purely structural explanations and recognizing that immune dysregulation is at the heart of DFU pathophysiology.

Hyperglycemia in diabetes creates oxidative stress, advanced glycation end products, and persistent nuclear factor κB activation, derailing the normal sequence of wound healing^2^, ^3^, ^4^ by promoting chronic inflammation and impairing tissue regeneration. Instead of transitioning from inflammation to proliferation, DFUs remain locked in a proinflammatory state. Macrophages fail to adopt a reparative phenotype, neutrophils overproduce reactive oxygen species, and proresolving signals are diminished. These wounds are, in many respects, a localized manifestation of diabetes as a systemic chronic inflammatory disease.

In acute healing, inflammation is swift and self-limiting, clearing pathogens and debris, giving way to repair. In DFUs, this is disrupted. Here, the four highlighted pathways offer mechanistic entry points to restore order. PGI_2_, a vasodilatory prostaglandin, suppresses nuclear factor κB via cyclic adenosine monophosphate-protein kinase A signaling.^5^ CXCR4 governs stem cell homing and leukocyte trafficking; pharmacologic inhibition can mobilize progenitor cells and enhance angiogenesis, but excessive blockade risks prolonging inflammation—underscoring the importance of context-specific modulation.^6^

The myeloid growth factor receptors CSF2RA and CSF3R connect directly to innate immune repair. Activation of the granulocyte-macrophage colony-stimulating factor (GM-CSF) receptor (CSF2RA) through perilesional GM-CSF injections has shown encouraging results, with rapid healing reported in small clinical series.^7^ Similarly, stimulation of the G-CSF receptor (CSF3R)—particularly in the setting of infected DFUs—has been associated with lower amputation rates in randomized trials.^8^

The takeaway is that treatment should move beyond broad, nonspecific anti-inflammatory approaches toward precision immunomodulation. Strategies such as enhancing PGI_2_ signaling, fine-tuning CXCR4 activity, and selectively engaging CSF2RA and CSF3R are not only promising experimental avenues but also mark a shift in mindset—one that seeks to restore the normal transition from inflammation to resolution in DFUs. Framing care through this immunologic lens may open the door to new, more effective management strategies for this challenging condition.

## Disclosures

None.
